# Identification of the metabolites of polybrominated diphenyl ether 99 and its related cytochrome P450s^☆^

**DOI:** 10.1016/S1674-8301(10)60032-2

**Published:** 2010-05

**Authors:** Huibin Dong, Ziyin Li, Xiaoming Man, Jingping Zhou, Huiyuan Lu, Shoulin Wang

**Affiliations:** Key Laboratory of Modern Toxicology of the Ministry of Education, School of Public Health, Nanjing Medical University, Nanjing 210029, Jiangsu Province, China

**Keywords:** polybrominated diphenyl ethers, hepatocytes, cytochroem P450s, metabolite, OH-PBDEs.

## Abstract

**Objective:**

To investigate the metabolites of polybrominated diphenyl ether 99 (BDE-99) and its related cytochrome P450s in an *in vitro* system.

**Methods:**

Rat primary hepatocytes were isolated and treated with BDE-99 for 24-72 h. Metabolites were then extracted from the hepatocytes and media, and detected by GC/MS. Several mRNAs of metabolic enzymes were also extracted from the same cells and the gene expression levels were determined using quantitative real-time PCR. In addition, selected recombinant cytochrome P450s (CYPs) were expressed in a bacurovirus/sf9 system, and these were further used to explore the metabolism of BDE-99 *in vitro*. The parent depletion approach was used for screening the ability of CYPs to eliminate BDE-99.

**Results:**

A reductively debrominated metabolite, BDE-47, and three oxidative metabolites, 2, 4, 5-tribromophenol, 5-OH-BDE-47, and 5′-OH-BDE-99, were identified from the BDE-99-treated rat hepatocytes, whereas no MeO metabolite was detected in the system. RT-PCR analysis showed that CYP 3A23/3A1, 1A2, and 2B1/2 were induced by BDE-99. Furthermore, using the heterological expressed CYP proteins in *in vitro* BDE-99 metabolism experiments we found that CYP1A2 and CYP3A4 showed the highest metabolic efficiency for BDE-99, with the metabolic clearance rates of CYP1A2 and CYP3A4 being 30.3% and 27.7%, respectively. CYP1A1 and CYP2A6 displayed relatively low clearance rates, while CYP2E1 seemed not to be associated with the BDE-99 metabolism.

**Conclusions:**

In our *in vitro* rat primary hepatocyte metabolism system, four metabolites of BDE-99 were identified, and CYP3A4 and CYP1A2 were demonstrated to be involved in the BDE-99 metabolism.

## INTRODUCTION

Polybrominated diphenyl ethers (PBDEs) are common flame retardants widely used in the manufacture of industrial and domestic equipments such as textiles, furniture, and electronic as well as electrical items to prevent fire and minimize fire damage. A number of rodent studies reported that PBDEs could have potential developmental neurotoxicity, developmental reproductive toxicity, and endocrine disruptive effects[1]. During the past decades, large amounts of PBDEs have been produced and applied, which has resulted in widespread contamination of the environment and accumulation of PBDEs in food webs. It is noteworthy that the concentrations of PBDEs in human and other body tissues have been increasing, with doubling times being approximately 4-6 years[2],[3]. BDEs 47, 99, 100, 153 and 154 are the major BDE congeners found in most human and environmental biota[2]. Many investigations on environmental samples indicate that the concentration of BDE-47 is approximately equal to or higher than that of BDE-99, with the concentration of BDE-153 at least 4-fold lower than that of BDE-99[4]–[6]. In contrast, in humans BDE-99 has the lowest concentration when compared to BDE-47 and BDE-153, or approximately at the same level as BDE-153[4],[7],[8]. Thus, BDE-99 appears to be metabolized to a greater extent than BDE-47 and BDE-153[9]–[12].

A number of studies on laboratory animal exposure to PBDE congeners reported that PBDEs could produce reductively debrominated metabolites and oxidative metabolites, such as hydroxylated BDE congeners (OH-BDE)[12],[13] and MeO-PBDEs which have been found in ringed seals, beluga whales and fish[14],[15]. Due to debrominated metabolites, high bioaccumulation and persistence[1] and increasing levels in the environment which are believed to contribute to the endocrine-disrupting effects of PBDEs, these compounds have become of increasing concern in recent years. Laboratory studies have confirmed that OH-BDE metabolites have greater adverse effects than PBDEs. For example, OH-BDEs have been shown to significantly affect aromatase activity in human adrenocortical carcinoma cells, whereas PBDEs had no effect[16]. Furthermore, OH-BDEs have an order of magnitude higher potency than PBDEs in their ability to compete with thyroid hormones for the binding sites on serum transporters[17]. Additionally, MeO-BDEs could be transformed to OH-BDEs[18]. Therefore, the information on the extent of formation of reductively debrominated metabolites and hydroxylated metabolites is critical to understand the complete risks associated with PBDE bioaccumulation and metabolism in humans and wildlife.

However, the metabolic processing of PBDEs is not clear. Cytochrome P450 monooxgenases, composing 70%-80% of all phase I xenobiotic-metabolizing enzymes, can metabolize a large number of endogenous compounds and are involved in many cellular functions[19]. Generally, CYP enzymes bind two atoms of oxygen, resulting in the formation of a water molecule together with the production of a metabolite. Usually when hydroxylation, dealkylation or oxidation occurs, ring-opening, and reduction can take place at the same time. The P450s families 1, 2, and 3 appear to be mainly responsible for the metabolism of a variety of exogenous and endogenous compounds. In animals and humans, CYPs can be found in virtually all organs including the liver, intestine, skin, lung, kidney, testis and brain. However, the liver is the predominant site of P450-mediated compound elimination, while the other tissues contribute to a much smaller extent to such compound elimination. Primary liver cells, containing abundant metabolizing enzymes, such as cytochrome P450s, are considered to be a better model to study the metabolism of PBDEs. But, there are limited opportunities to study these cells because primary human hepatocytes are difficult to obtain. Therefore, primary rat hepatocytes are used instead to establish a system for PBDEs metabolism. To date, there are several studies on PBDE metabolism in human microsomes and hepatocytes[20]. The up regulation of the CYP genes and the formation of several oxidative metabolites of PBDEs suggested a key role of CYP-mediated metabolism[21]. However, so far, little information on BDE congener metabolism in human cytochrome P450s has been provided.

In the present study, we established an *in vitro* model using primary rat hepatocytes and recombinant cytochrome P450s to detect whether reductively debrominated, OH and/or MeO metabolites of BDE-99 would be produced, which cytochrome P450 was involved in the BDE-99 metabolism, and compared the BDE-99 metabolic activities of cytochrome P450s. The study has the potential to provide a better understanding of the pathways through which PBDEs are metabolized in humans.

## MATERIALS AND METHODS

### Chemicals and reagents

The test compounds BDE-47 (98% purity) and BDE-99 (99% purity) were purchased from Wellington Laboratories (Guelph, Ontario, Canada). BDE-17, BDE-28, BDE-77, 3-OH-BDE-47 , 5-OH-BDE-47, 6-OH-BDE-47, 5′-OH-BDE-99, 6′-OH-BDE-99, 5′-MeO-BDE-99 and 6′-MeO-BDE-99 were obtained from AccuStandard (New Haven, CT, USA). All their purities were greater than 97%. Hepatocyte culture medium, unsupplemented Grace Insect Cell Culture Medium and SF900 II SFM, antibiotics, collagen-coated culture plates, Sf9 insect cells, Bac-to-Bac baculovirus expression system, and Cellfectin® reagent were from GIBCO (Invitrogen Corporation, USA). NADPH, ferric citrate, 5-ALA and monoclonal anti-CYP1A1, CYP1A2, CYP2A6, CYP3A4 and CYP2E1 antibodies were purchased from Sigma-Aldrich (St. Louis, MO, USA).

### Isolation of primary rat hepatocytes

Primary rat hepatocytes were isolated from three individual male Sprague-Dawley CD rats (body weight 220-250 g) obtained from Laboratory Animal Center of Nanjing Medical University, using the two-step in situ collagenase perfusion method[22]. Then the hepatocytes were purified by percoll density gradient separation and washed twice before being resuspended in the attachment medium. Cells viability exceeded 90% as determined by trypan blue exclusion. For the cell culture, the isolated hepatocytes were seeded in Type I collagen-coated 12-well plates at a density of 150, 000 cells/cm^2^ in Williams' Medium E containing 5% fetal calf serum, 100 U/mL penicillin and 100 µg/mL streptomycin, and then incubated in a humidified atmosphere of 5% CO_2_ in air at 37°C. After 2-3 h incubation, the unattached cells were poured off and the medium was replaced with 1 mL HepatoZYME-SFM supplemented with 100 U/mL penicillin, 100 µg/mL streptomycin, 20 µg/L EGF and 20 µg/L HGF.

### Hepatocyte treatment with BDE-99

All the hepatocyte batches were exposed to BDE-99 at a nominal concentration of 10 µM, equivalent to 10 nM of compound per well. A stock solution of BDE-99 which was 200 times the final concentration in tissue culture medium was prepared in DMSO and stored at -20°C. Separate 12-well plates were used for the metabolism and gene expression analysis. To produce metabolites of BDE-99, the hepatocytes were treated once every 24 h for 3 days to take advantage of the increased activity of cells and potential increase in metabolite formation. The hepatocytes cultured with medium without BDE-99 were set up as controls. During medium exchange of the hepatocytes, we collected and pooled the contents from each well. After incubation, the hepatocytes were removed from the wells using 1 mL methanol to disrupt cell membranes. The contents were subsequently transferred to clean glass test tubes for extraction. For the gene expression analysis, the rat hepatocytes were exposed to 10 µM BDE-99 for 24 h. Wells treated with media containing aliquots of DMSO were set up as controls.

### Gene expression analysis

The expression of several genes that encode potential biotransforming enzymes, such as CYP1A2, CYP2B1/2, CYP3A23/3A1, GSTM1 and GSTP1, were determined using quantitative RT-PCR, and GAPDH was used as a reference to calculate the expression levels. Primers were designed using Primer 5.0 software, and were synthesized by the Invitrogen Corp., Shanghai. The PCR primer sequences are shown in ***Table 1***. When the total RNA and their corresponding cDNA were prepared, the RT-PCR was performed on the ABI PRISM 7900HT Sequence Detection System. The temperature profile was set up as follows: 50°C for 2 min, 95°C for 10 min followed by 40 cycles of 95°C for 15 s, and finally 60°C for 1 min extension. At the end of each step, the fluorescence intensity of evagreen was read on the 7900 system. Melting curve analysis was created following the final PCR cycle to confirm the presence of a single PCR product. Relative gene expression was obtained by the method described previously (Relative expression = 2^−ΔΔCt^)[23].

**Table 1:** Primer sets for quantitative RT-PCR analysis.

Gene	Accession No	Primers sequence
CYP1A2	NM_012541	F: 5′ TTTGGAGCTGGATTTGAAACAGT 3′
		R: 5′ TCATGAATCTTCCTCTGCACCTT 3′
CYP2B1/2	NM_37134	F: 5′ CCCAATGTTTGGTGGAGGAA 3′
		R: 5′ CTGTGATGCACTGGAAGAGGAA 3′
CYP3A23/3A1	NM_173144	F: 5′ CAGCAGCACACTTTCCTTTGTC 3′
		R: 5′ CTCCTCCTGCAGTTTCTTCTGTGTA 3′
GSTM1	NM_017014	F: 5′ CGACGCTCCCGACTATGACA 3′
		R: 5′ CACGAATCCGCTCCTCCTCT 3′
GSTP1	NM_012577	F: 5′ GCACCTGGGTCGCTCTTTA 3′
		R: 5′ GGGCCTTCACATAGTCATCCTT 3′
GAPDH	NM_017008	F: 5′ CATAGACAAGATGGTGAAGGTCGG 3′
		R: 5′ GTCCCACTTTGTCACAAGAGAAGGC 3′'

### Recombinant expression and identification of human cytochrome P450s

All the proteins were expressed in a Bac-to-Bac baculovirus expression system (Invitrogen Corp., USA) following the manufacturer's instructions. Briefly, the CYP1A1 (NM_000499), CYP1A2 (NM_000761), CYP2A6 (NM_000762), CYP2E1 (NM_000773) and CYP3A4 (NM_017460) cDNA were first transformed to the recombinant bacmid, and subsequently transfected into the Sf9 cells to produce recombinant baculovirus particles, and further to get the baculoviral stock which was finally used to express CYPs proteins in the culture medium containing 0.1 µM ferric citrate and 0.1 µM 5-ALA. After being incubated for 72 h, the infected Sf9 cells were harvested and the microsomes were prepared by sonication using an Ultrasonic Processor (CPX130, Cole-Parmer Scientific Instruments, USA) and differential centrifugation. The protein expressed from the blank vector was used as a negative control. The prepared microsomes were stored at -80°C prior to usage.

The protein expression was determined using immunoblotting. Briefly, the microsomal proteins were separated by SDS-polyacrylamide gel electrophoresis and transferred to a nitrocellulose sheet, and then incubated with a monoclonal antibody against human CYP1A1, CYP1A2, CYP2A6, CYP2E1 and CYP3A4 as the primary antibody. This was followed by the binding of a secondary antibody conjugated with horseradish peroxidase, and the immunoblots were visualized by ECL detection according to the manufacturer's protocol. Microsomal proteins were diluted to 2 mg/mL with the reaction buffer. P450 content was determined by reduced CO-differential spectrum following the method described elswhere[24]. The difference in spectrum absorption was measured by scanning from 500 nm to 400 nm using a DU® 800 UV/visible spectrophotometer (Beckman Coulter, USA).

### Preparation of metabolic clearance reactions by human cytochrome P450 isozyme

Each human P450 was screened for its ability to oxidize and eliminate BDE-99 from the assay mixture. The total volume of mixture was 0.5 mL and contained 0.1 M Tris-HCl (pH 7.4), 10 pmol/mL of P450, 50 pmol/mL of P450 reductase and 1 mg/mL NADPH. After the assay mixture was preincubated for 10 min at 37°C, the reaction was initiated by addition of 2 µl of 5 mM BDE-99 as stock solutions to yield a final concentration of 20 µM. Assays were carried out for 20 min and quenched by transferring tubes to an ice bath. To determine the relative rate of metabolism of BDE-99, human cytochrome P450 isozymes were incubated with cold BDE-99 as described above for 20 min. The protein expressed from the blank vector was used as a control reaction, to verify that metabolism was enzymatic. Each incubation was conducted in triplicate (n = 3) and samples were then frozen and stored at -20°C pending analysis.

### Sample extraction

Hepatocytes and media were extracted using methods developed for the extraction of phenolic and neutral compounds from serum[25]. Briefly, samples were first spiked with one internal standard, BDE-77, and extracted using methyl-tert-butyl ether/hexane:1/1(v/v). Lipids were removed from the extracts with concentrated sulfuric acid, and then the neutral and phenolic compounds were separated using a basic aqueous solution of potassium hydroxide. The phenolic fraction was derivatized with acetonitrile / methanol / water / pyrimidine: 5/2/2/1(v/v), then methyl chloroformate was added to produce methyl formate BDE congeners. After derivatization, samples were evaporated to complete dryness using a slow stream of nitrogen and reconstituted in hexane. Each human P450s assay sample was added into one internal standard BDE-77 before extraction. Proteins were denatured with 0.5 mL 6 M hydrochloric acid, and then 3 mL hexane was added for extraction. This step was repeated three times. The extraction was evaporated to complete dryness using a slow stream of nitrogen and reconstituted in 0.5 mL acetonitrile. Rates of elimination were determined by comparing the concentration of parent compound before and after the reaction.

### Metabolites analysis by GC/MS.

The underivatized and derivatized extracts were analyzed using a GC/MS system (Trace DSQ ThermoFisher Scientific, USA) with electron impact (EI) ionization. Separation was performed on a DB-5MS column (30 m×0.25 mm i. d. ×0.25 µm thickness) (Agilent Technologies, Inc., USA). The inlet temperature was 260°C, and the injection was performed in pulsed splitless mode. The oven initial temperature was 110°C and was held for 1 min. The temperature was ramped to 210°C at 12°C/min, and a second ramp was performed to 250°C at 4°C/min and was held for 10 min. Finally, the oven was ramped to 300°C at 20°C/min and this step was held for 10 min to give a total run time of 32.83 min. The auxiliary temperature for the MSD was 280°C. A full scan to determine initial mass to charge (m/z) ratios was performed with the MSD from m/z 70 to 1,000, after which selected ion monitoring (SIM) was used for GC/MS analysis. PBDEs and OH-BDEs were monitored using the m/z responses of 79 and 81 (bromide ions). Quantification and qualifier ions are provided in ***Table 2***.

**Table 2:** Quantification and/or qualification ions for selected ion monitoring of parent compounds and metabolites.

Compound	Molecular ion (m/z)	Fragment ions (m/z)	Retention time (min)
BDE-47	486	326,486	19.10
BDE-77	486	281,486	20.80
BDE-99	564	404,564	24.40
2, 4, 5-tribromophenol	390	329,344	9.82
5-OH-BDE-47	560	356,420	23.01
5′-OH-BDE-99	639	419,436	25.97

### Quality control

Recovery of the surrogate standard BDE-77 averaged 87%±13%. All the solvents were checked for any potential contamination of the PBDE analysis before being used. The samples were analyzed in duplicates using procedure blanks and limits of detection (LODs) were defined as three times of the SD of the laboratory controls. For congeners not detected in the blanks, the LOD was set at the instrumental limit of quantification.

### Statistical analysis

BDE-99, metabolites, and gene expression data were expressed as mean±SD. Differences between BDE-99 and control groups were analyzed using paired Student's t-test. The RT-PCR was performed in duplicate for three primary rat hepatocytes. All statistical analyses were performed using SPSS software (Version 11.0, SPSS Inc., USA). The P-value reported was two-sided and value of *P* < 0.05 was considered statistically significant.

## RESULTS

### Identification of metabolites in primary hepatocytes treated with BDE-99

Before the metabolites were identified, the recovery rate of BDE-99 was determined using GC/MS of the neutral fractions of extractions collected from the hepatocytes. First, the cells were treated with the BDE-99 at the initial concentration of 10.21±0.50 nmol/well. After the 24-h-incubation, 8.83±0.16 nM of BDE-99 was recovered in the hepatocyte wells, which meant that about 13.5% of the BDE-99 mass was unrecovered. Based on these results, it was speculated that the amount of unrecovered mass of BDE-99 was likely to be attributed to the metabolic production of its metabolites. As discussed above, two kinds of BDE-99 metabolites, reductively debrominated metabolites and oxidative metabolites, were considered in the metabolism.

**Fig. 1:**
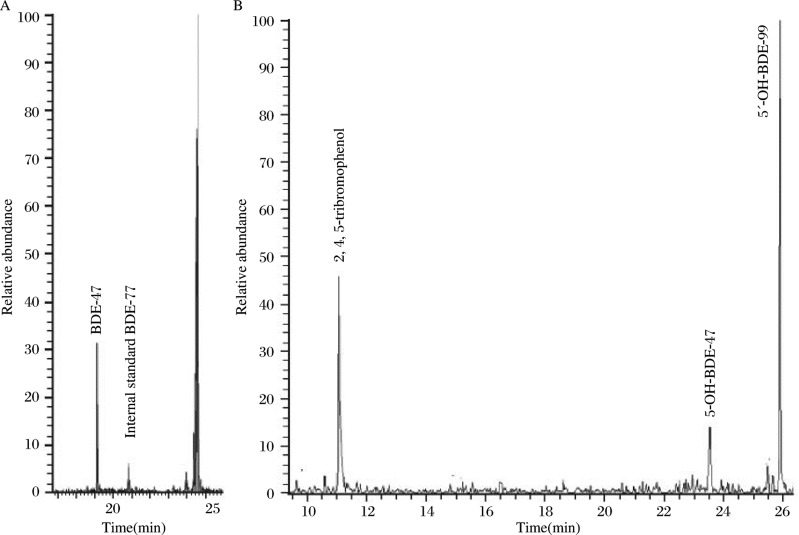
Chromatogram of BDE-99 metabolites from the rat hepatocytes incubated with 10 µM BDE-99. A: The GC/MS chromatogram (m/z 79 and 81) of the neutral fraction, identifying a reductively debrominated metabolite BDE-47. BDE-77 was used as internal standard. B: The GC/MS chromatogram (m/z 79 and 81) of the derivatized phenolic fraction, identifying the three metabolites 2, 4, 5-tribromophenol, 5-OH-BDE-47, 5′-OH-BDE-99.

As shown in ***Fig. 1A***, one reductively debrominated metabolite but no MeO-BDE-99 metabolite was observed in the neutral extracts isolated from the hepatocytes exposed to BDE-99. BDE-17, BDE-28 and BDE-47 were used as the reference compounds to detect the tribromodiphenyl ether and/or tetrabromodiphenyl ether metabolites, and the molecular mass was analyzed by full-scan GC/EI-MS to detect other debrominated metabolites. Consequently, comparing the retention time and the molecular ion and ion fragment clusters of metabolite with the commercial standards, a tetrabromodiphenyl ether metabolite, BDE-47, was found in all hepatocytes exposed to BDE-99. The average concentration was 215.9±35.8 pmol/well. Unfortunately, when using 5′-MeO-BDE-99 and 6′-MeO-BDE-99 as references and a strategy similar to the above, no MeO metabolite was determined in our experiments. These results indicated that the reductive debromination was possibly a substantial metabolic pathway in rat liver tissue.

On the other hand, three kinds of oxidative metabolites were observed in the extractions from the phenolic fraction of hepatocytes exposed to BDE-99 in all rat liver cells ***(Fig 1B)***. Based on the molecular ion clusters in the GC/MS full scan, these three metabolites were found to contain three, four and five bromine atoms, respectively. The commercial standards such as 2, 4, 5-tribromophenol, 3-OH-BDE-47, 5-OH-BDE-47, 6-OH-BDE-47, 5′-OH-BDE-99 and 6′-OH-BDE-99 were used as references which were derivatized under the same conditions as the phenolic extracts. At the same time, these metabolites were compared with the commercial standards for the retention time, the molecular ion and ion fragment clusters. Finally, they were identified as a tribromophenol of 2, 4, 5-tribromophenol, a monohydroxylated tetrabromodiphenyl ether metabolite of 5-OH-BDE-47, and a monohydroxylated pentabrominated diphenyl ether metabolite of 5′-OH-BDE-99, respectively ***(Fig. 1B)***. However, due to the difficulties of the qualitative analysis, they should be quantified in a further study.

### Cytochrome P450 mRNA expression in primary hepatocytes induced by BDE-99.

**Fig. 2:**
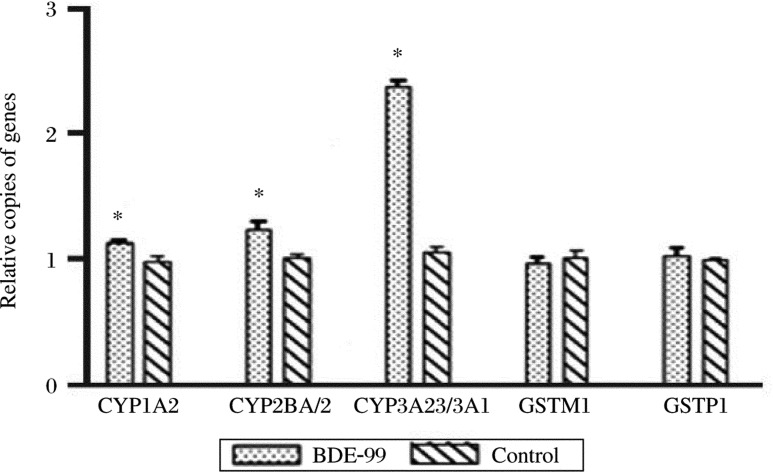
mRNA expression of several genes encoding for potential metabolic enzymes in rat hepatocytes exposed to BDE-99. cDNA (0.1 µg) for each gene was used to determine the mRNA expression level by real-time RT-PCR. Transcript numbers were compared between the negative control (no BDE) and each treatment (mean±S.D, *n* = 3), *P* < 0.05.

To investigate the potential involvement of several metabolizing enzymes in the BDE-99 metabolism, the mRNA expression of CYP1A2, CYP2B1/2, CYP3A23/3A1, GSTM1 and GSTP1 were determined from the three primary rat hepatocyte preparations using the same BDE-99 treatment as above. The relative transcript number of each target gene in the treated cells was calculated by comparing with the control cells. As shown in ***Fig. 2***, the expression of CYP1A2, CYP2B1/2, and especially CYP3A23/3A1 in the BDE-99 group were significantly higher than the control (*P* < 0.05), and the average percentage increases were 15%, 22% and 124%, respectively. However, there were no differences between the treated cells and the control cells for GSTM1 and GSTP1. These results indicated that several CYPs, especially the CYP3A subfamily, might be involved in the BDE-99 metabolism.

### Metabolic clearance efficiency of BDE-99 by recombinant expressed human cytochrome P450 isozyme

**Fig. 3:**
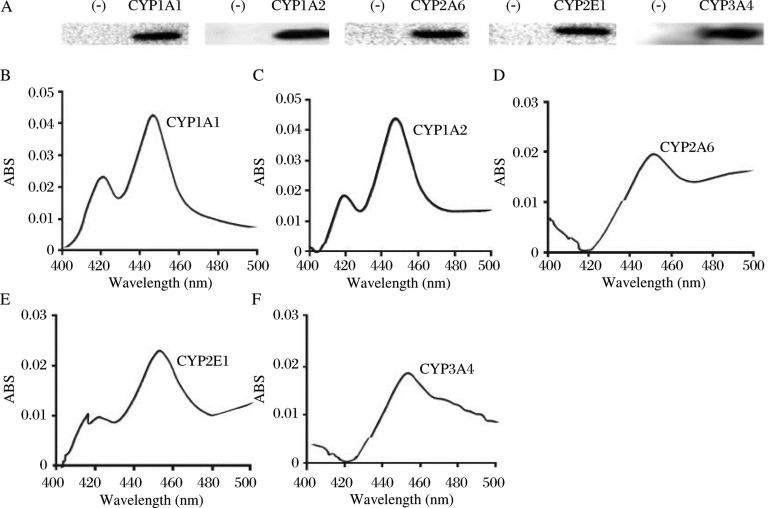
Identification of heterologous expression of recombinant CYP1A1, CYP1A2, CYP2A6, CYP2E1 and CYP3A4 cDNAs in Sf9 cells. A: 0.5 µg microsomal protein of each of the CYPs were loaded to detect the protein expression and purity by immunoblotting. The microsomes from the Sf9 cells infected with the blank vector without any CYP cDNA were used as negative control (-). The microsomal proteins of CYP1A1 (B), CYP1A2 (C), CYP2A6 (D), CYP2E1 (E), CYP3A4 (F) were adjusted to 2 mg/mL for CO-differential assay, and each protein showed the characteristic P450 peak.

Recombinant expressed human CYP1A1, CYP1A2, CYP2A6, CYP2E1 and CYP3A4 were used to assess the BDE-99 metabolism *in vitro*, and the proteins were identified using immunoblotting and CO-differential assay. The results showed that all the single proteins displayed a specific band with the expected molecular weight, whereas no bands were found in the blank vector cells ***(Fig. 3A)***. Accordingly, all the single CYP proteins showed a characteristic peak at 450 nm ***(Fig. 3B)***, with the P450 content ranging from 0.8 to 0.31 nmol/mg. The results above indicated that all the recombinant cDNAs were successfully expressed for the functional P450 enzymes which were critical for the BDE-99 metabolism. As shown in ***Table 3***, both the human CYP1A2 and CYP3A4 eliminated BDE-99 at a significantly higher rate than the other selected CYPs, with metabolic clearance rates of 30.3%±6.5% and 27.7%±5.1%, respectively. The metabolic efficiency of CYP1A1 and CYP2A6 with BDE-99 were similar and lower than CYP1A2 and CYP3A4. They converted 14.5%±3.6% and 11.6%±2.3% of the BDE-99, respectively. However, similar to the vector protein, the CYP2E1 did not contribute to any metabolism of BDE-99. These results indicated there were very large differences among the different CYPs in the metabolism of BDE-99. CYP1A2 and CYP3A4 might be metabolic enzymes with higher efficiencies, which was consistent with the results from the CYP mRNA expression study in rat hepatocytes induced by BDE-99.

**Table 3:** Metabolic clearance rate of BDE-99 by human cytochrome P450 isoforms.

Isoform	Clearance rate of BDE-99 (%)
CYP1A1	14.5±3. 6 ^a^
CYP1A2	30.3±6.5
CYP2A6	11.6±2.3
CYP2E1	N.S. ^b^
CYP3A4	27.7±5.1
Control	N.S.

^a^Percentage parent BDE-99 metabolized during a 20-min incubation; mean ±SD, *n* = 3. ^b^N.S. : no significant difference, *P* < 0.05.

## DISCUSSION

The present study demonstrated that BDE 99 could be metabolized by rat liver cells primarily through reductive and oxidative pathways which produced the reductively debrominated metabolite of BDE-47 and oxidative metabolites of 2, 4, 5-tribromophenol, 5-OH-BDE-47 and 5′-OH-BDE-99. The results of the production of the reductively debrominated metabolites were supported by several *in vivo* studies and one *in vitro* study using liver subcellular fractions that showed significant reductive debromination of BDE congeners 99, 183, and 209 in fish, rodents, and birds[26]–[28]. Although one study showed no reductively debrominated metabolites produced in human hepatocytes exposed to either BDE-99 or BDE-209[21], the possible reason was that it might be associated with species-specific differences in the metabolism of BDE congeners[29]. Due to the poor thermal stability and low volatility, the hydroxylated BDEs could not be detected in their underivatized form by GC/MS analysis. Therefore, phenolic metabolites were derivatized to methyl formate analogs, suitable for GC/MS analysis to improve the detection limits. In the current study, we still found several oxidative metabolites from the phenolic fraction of the extraction which were consistent with several previous studies that identified oxidative metabolites of BDE-99 in rat and mouse urine[12],[13]. This was similar to a study that found 2, 4, 5-tribromophenol, one mono-tetra-OH BDE, and two mono-OH-BDE-99 metabolites in the feces of rats exposed to BDE-99 *in vivo*[9]. Because 5′-OH-BDE-99 was found at higher concentrations of BDE-99 than pentahydroxy-BDE metabolite in the human hepatocytes[21], it was speculated that oxidative metabolites, especially 5′-OH-BDE-99, were sensitive indicators of BDE-99 exposure. In contrast to some other studies[30],[31], none of the MeO metabolites in the neutral extracts was observed in our study, which indicated that the MeO metabolite was not produced or the exposure period was not long enough. Compared with the reductively debrominated metabolite, the MeO might not be in a substantial metabolic pathway in rat liver tissue. To some extent, this difference might be associated with the fact that MeO-PBDEs could be transformed to OH-PBDEs *in vitro* by microsomal incubation[18],[30].

On the other hand, several CYP genes (CYP1A2, CYP2B1/2, especially CYP3A23/3A1) were found to be up-regulated in BDE-99 exposed primary rat hepatocytes, which was confirmed by our *in vitro* metabolism study that found CYP1A2 and CYP3A4 were the most efficient enzymes metabolizing BDE-99. The results demonstrated that CYP1A2, especially CYP3A4, might be the specific metabolic enzymes for BDE-99. Some similar studies showed that CYP1A2 and CYP3A4 could be induced in human hepatocytes exposed to BDE-99 and BDE-209[21], and these results were also supported by the study using human MCF-7 breast cancer cells exposure to PBDEs[32]. Therefore, liver CYP isoenzymes may serve as a sensitive biomarker for long-term exposure to polyhalogenated hydrocarbons[33]. Metabolism of BDE-99 by human P450 isoforms might provide a clear understanding of the role of P450s in PBDEs metabolism. Using a parent depletion approach, CYP3A4 and CYP1A2 showed much higher efficiencies in metabolizing BDE-99 than CYP1A1 and CYP2A6, whereas CYP2E1 seemed not to be related to the metabolism of BDE-99. As we known, approximately 30% and 13% of total P450 in human liver are composed of CYP3A families and CYP1A2, respectively[34], which leads to the assumption that CYP1A2 and CYP3A4 are the most likely isoforms to play a major role in metabolizing PBDEs in human liver. Being different from CYP3A4 and CYP1A2, CYP1A1 is essentially an extrahepatic enzyme that is present predominantly in the intestine. The lack of CYP1A1 induction by highly purified PBDEs in various rodent, monkey and human cell systems *in vitro*[35],[36] implies that CYP1A1 was not the major metabolic enzyme for PBDEs. The results also showed the CYP2A6 and CYP2E1 had much lower metabolic efficiencies for BDE-99, which also indicated that CYP2A6 and CYP2E1 did not play a major role in the metabolism of PBDEs.

While previous studies have assessed the metabolism and disposition of PBDEs in rats and mice[9],[13], our study is one of several recent reports that describe the qualitative metabolism of BDE-99 by primary hepatocytes[21],[36]. Clearly, primary rat hepatocytes provide not only a more economical and faster system than the overall animal experiments, but also a more physiologically relevant and sensitive system than cell lines[37],[38]. Although ethical considerations may prevent PBDEs studies in some mammals, *in vitro* assays can provide a viable alternative method by which the environmental contaminant (s) of interest can be incubated with prepared human P450 enzymes and metabolic activity can be examined by monitoring depletion of the parent compound (s) and/or formation of metabolites. Besides, since *in vitro* assays can not represent what actually happens *in vitro*, when there are many steps (e.g. reductive reactions, phase II reactions) involved in the potential biotransformation of a single congener, one step through which CYP-mediated oxidative biotransformation can be thoroughly studied[29].

Biotransformation of PBDEs by their specific cytochrome P450s is definitely considered to play a critically important role in their induced toxicity. Therefore, finding and the identification of PBDE metabolites and potential biotransformation pathways in humans will assist efforts to better understand not only the factors regulating the disposition and retention of these contaminants, but also the potential toxicity of PBDE metabolites. Because the mechanism(s) of toxicity are not clearly understood, it is important to consider the different toxicokinetic parameters associated with each congener when assessing the risk to human health of individual PBDE congeners as well as the commercial PBDE mixtures.
